# The Microbiota and Immune System Crosstalk in Health and Disease

**DOI:** 10.1155/2018/2912539

**Published:** 2018-04-22

**Authors:** Rossella Cianci, Danilo Pagliari, Ciriaco A. Piccirillo, Jörg H. Fritz, Giovanni Gambassi

**Affiliations:** ^1^Institute of Internal Medicine, “A. Gemelli Hospital”, Catholic University of the Sacred Heart, Rome, Italy; ^2^Department of Microbiology and Immunology and Program in Infectious Disease and Immunity in Global Health and Centre of Excellence in Translational Immunology (CETI), Research Institute of the McGill University Health Centre, McGill University, Montréal, QC, Canada; ^3^Department of Microbiology and Immunology and Department of Physiology and McGill University Research Center on Complex Traits (MRCCT) and Centre of Excellence in Translational Immunology (CETI), Research Institute of the McGill University Health Centre, McGill University, Montréal, QC, Canada

As gut microbiota, the whole intestinal microbial collection is indicated; it includes trillions of microorganisms and at least 1000 different bacterial species [1]. The microbiome, instead, is the collection of the whole genome sequences of those microorganisms, and it consists of more than 5,000,000 genes. In physiological conditions, a perfect *equilibrium* among commensals and pathogens maintains intestinal homeostasis. Any imbalance precipitates a pathological state known as “gut dysbiosis” [2].

Gut microbiota modulates host physiology and metabolism through different mechanisms. A close relationship exists with sex-based hormones. On one hand, gut microbiota can induce hormonal changes leading to inflammation, and, on the other hand, hormone levels themselves shape microbiota composition. Variations in gut microbiota composition may be responsible for modifications of the host hormonal axis but also in modulation of the immune system function. Recently, a germ-free NOD mice model demonstrated that the colonization by certain commensal bacteria induced an elevation in testosterone levels in males versus females [3]. Other evidences have shown that antibiotics may be responsible for alterations in sterol metabolism-associated gene expression and also in T cell differentiation. This hormone-dependent modulation of gut microbiome explains the different susceptibilities to disease between men and women [4].

Gut microbiota is also strictly linked to the chronological age of each individual. In fact, each stage of human life is characterized by a specific intestinal microbial composition. An epochal turn in human life is set up at birth when sterility of the whole body is challenged by bacterial colonization in the birth canal. During adulthood, gut microbiota becomes stable and in *equilibrium* with the host, then it undergoes significant changes at older ages that correlate with residual physical function and life settings [5].

Diet and food supplements exert a great impact on gut microbial composition and its variability through time. In Western countries, a high-fat diet is a risk factor for disorders, such as obesity, metabolic syndrome, and diabetes all of which are associated with profound modifications of gut microbiota composition [6]. Interaction between food and gut microbiota is also finely tuned by our circadian clock. The disruption of the physiological circadian rhythm increases the likelihood of a gut dysbiosis, possibly contributing to the pathogenesis of several metabolic and inflammatory diseases, including diabetes, inflammatory bowel diseases, and even cancer [7].

In more recent years, the concept of a “gut-brain axis” has been introduced. The endocrine system may be modulated at the intestinal level in a sort of neuro-entero-endocrine system. This system interacts with the immune system at the mucosal level in order to maintain a homeostasis but also to enhance defense against microbial invasion in pathological states. As a result, modifications of microbiota composition may be associated to several disorders of the nervous system, including neuropsychiatric, neurodegenerative, and neuroinflammatory disorders [8].

It is well established that the gut microbiota is in close interaction with the intestinal mucosal immune system. Indeed, the intestinal mucosa may be considered as an immunological niche as it hosts a complex immune-functional organ comprised by T cell subpopulations and their related anti- and proinflammatory cytokines, as well as several other mediators of inflammation, in addition to the microbiota. Both innate and adaptive immune systems are involved in the gut immunological niche [9]. Several barriers protect the immunological niche from the invasion of pathogens. First, there is a mechanical barrier provided by the mucus layer with antimicrobial peptides, such as alpha-defensins, and secretory IgA and by enterocytes that possess tight intercellular junctions. Neutrophils and innate lymphoid cells (ILCs) represent another cellular defensive line. Finally, macrophage-dendritic cell- (DC-) and lymphocyte-associated pattern recognition receptors, such as Toll-like receptors (TLRs) and NOD-like receptors (NLRs), provide a further barrier bridging the activation of both innate and adaptive immune cells. Activated by bacteria, all of these mechanisms determine a proinflammatory adaptive immune response that involves several T cell subpopulations, such T helper 1 and 17 (Th1 and Th17 cells), and their related cytokines, such as IL-1, IL-2, IL-15, IL-17, IL-22, and IL-23 [10].

Any modification in the balance between microbiota and gut immunological niche components may trigger infectious, inflammatory, and endocrine diseases [11], such as metabolic liver disorders, inflammatory bowel diseases, pancreatic disorders, autoimmune diseases, and aging [12, 13]. This holds true also for cancer. There is evidence that gut dysbiosis favors the formation of gastrointestinal tumors—colorectal, gastric, liver, and pancreatic, and it might also be responsible for the modulation of antitumor response during chemotherapy and immunotherapy [14].

In this special issue, a series of manuscripts highlighting the relevance of gut microbiota and immune system cross-talk in several disease states are collected.

S. Bibbò et al. report that leaks in the intestinal mucosal barrier lead to the translocation of bacterial products into portal circulation stimulating innate immunity via TLRs. This translates into a release of proinflammatory cytokines causing chronic liver inflammation, heralding diseases like nonalcoholic fatty liver disease (NAFLD) and nonalcoholic steatohepatitis (NASH).

A similar mechanism seems involved in the pathogenesis of various pancreatic disorders. In their article, D. Pagliari et al. summarize the evidence linking a disruption of the intestinal mucosal barrier to several benign and malignant disorders, such as pancreatitis, diabetes, and pancreatic cancer. A specific microbial profile for each pancreatic disease seems established such that novel tools for prevention and early diagnosis are now underway.

A microbiota is present also in systems other than the gastroenteric tube, and these microorganisms exert additional physiological functions. The skin is not only a mechanical barrier to the external world but it also plays a crucial role in maintaining homeostasis between pathogen-related immune responses and the external environment. F. Abdallah et al. examine the role of skin microbiota in the pathogenesis of immune-mediated disorders, such as atopic dermatitis and *acne vulgaris*.

Similarly, gut microbiota may be implicated in the induction and in the maintenance of local and systemic inflammation in autoimmune diseases. F. Biscetti et al. review the role of the microbiota-related activation of the conserved DNA-binding protein high-mobility group box-1 (HMGB1) in synovial inflammation of patients with rheumatoid arthritis. Since HMGB1 can be activated by the Gram-negative lipopolysaccharide (LPS) via the TLR-4 pathway, it may turn out to be a possible target for novel therapeutic approaches.

In several instances, xenobiotics have been suggested to interfere with inflammatory pathways in the intestinal mucosa eliciting an anti-inflammatory and immune-modulating action. D. Pagliari et al. review the interaction of rifaximin with several components of the gut immunological niche that explain its ability to reduce intestinal dysbiosis and to inhibit the release of proinflammatory mediators.

Interestingly, rifaximin has been successfully employed in patients with colchicine resistance familial Mediterranean fever (FMF). In their article, E. Verrecchia et al. demonstrate that in patients with innate immunity hypersensitivity, such as FMF, gut dysbiosis worsens the clinical manifestations of the disease. Conversely, treatment with rifaximin increases the efficacy of colchicine and ameliorates FMF.

Indeed, other xenobiotics can be involved in the modulation of inflammation. J. Lu et al. explore the anti-inflammatory properties of columbianetin, derived from the root of a Chinese herb *Radix Angelicae Pubescentis.* In LPS-stimulated human peripheral blood mononuclear cells, the molecule suppresses the expression of several NF-*κ*B-mediated proinflammatory cytokines.

New approaches to the treatment of human metabolic and behavioral disorders are being tested. C. Colica et al. have conducted a clinical study on obese and nonobese patients where they administered a probiotic formulation containing a cocktail of *Lactobacillus* spp., *Bifidobacterium* spp., and *Streptococcus* spp. This formulation has produced positive effects on obesity but also on psychological distress.

Finally, changes in gut microbiota have been linked to the chronic, low-grade inflammation associated with aging, named “inflamm-aging.” In their article, A. Picca et al. examine how such microbial changes modulate the inflammatory pathways involved in the development of sarcopenia and cachexia. These signaling pathways may provide novel targets for future therapeutical interventions.

The overall aim of this special issue is to collect and summarize current knowledge about the complex interplay among gut microbiota, immune system, mediators of inflammation, and xenobiotics in health and during diseases. We trust that there is abundant food for thoughts.
